# Enabling artificial intelligence-driven blind joint ultrasound self-scans in hemophilia: an annotation protocol

**DOI:** 10.1016/j.rpth.2026.107019

**Published:** 2026-10-13

**Authors:** Daniel Michelsanti, Nima Teimouri, Andreas Heindl, Karen Strike, Roberta Gualtierotti, Andrea S. Doria, Carlo Martinoli, Johannes Roth

**Affiliations:** 1 AI & Analytics, Novo Nordisk A/S, Søborg, Denmark; 2 Hamilton Niagara Hemophilia Treatment Center, McMaster Children’s Hospital, Hamilton Health Sciences, Department of Rehabilitation Science, McMaster University, Hamilton, Ontario, Canada; 3 Angelo Bianchi Bonomi Hemophilia and Thrombosis Center, Fondazione IRCCS Ca’ Granda Ospedale Maggiore Policlinico, Department of Pathophysiology and Transplantation, University of Milan, Milan, Italy; 4 Department of Diagnostic & Interventional Radiology, The Hospital for Sick Children, Medical Imaging Department, University of Toronto, Toronto, Canada; 5 Dipartimento di Scienze della Salute, University of Genoa, Genoa, Italy; 6 Children’s Hospital of Central Switzerland, Luzern, Switzerland

**Keywords:** artificial intelligence, delivery of health care, hemophilia A, hemophilia B, ultrasonography

## Abstract

**Background:**

Early detection and management of joint bleeding are critical for preventing long-term joint damage in people with hemophilia. However, current practices often require healthcare professionals to assess bleeding, limiting patient autonomy and potentially leading to delayed or unnecessary treatment. Current literature primarily focuses on clinical protocols for ultrasound (US) imaging but lacks annotation protocols tailored for training artificial intelligence (AI) systems.

**Objectives:**

To propose a structured annotation protocol to support the development of an AI-driven, ultraportable US system that enables people with hemophilia to perform self-guided scans of their joints, focusing specifically on the posterior recess of the elbow, anterior subquadricipital recess of the knee, and anterior recess of the ankle.

**Methods:**

The annotation protocol defines eight criteria to assess the appropriateness of US images and five levels for region-of-interest clarity. A pool of six readers rated US videos of elbows, knees, and ankles from 100 healthy subjects, and their annotations were analyzed using the intraclass correlation coefficient (ICC).

**Results:**

The application of the proposed protocol demonstrates moderate to good agreement based on the ICC when assessing the reliability of single ratings from individual readers (ICC(2,1) = 0.7643), with the knee exhibiting the highest reliability. When considering average ratings across multiple readers, we observe overall excellent reliability, with an ICC of 0.9075.

**Conclusion:**

This annotation protocol supports the development of future AI-guided image-acquisition systems for joint self-scanning, marking a significant step toward at-home joint assessment for people with hemophilia. Future research should address the evaluation of the AI guidance system, patient-performed scanning, and diagnostic accuracy for hemarthrosis.

## Essentials

• Guidelines for bleed detection in hemophilia require healthcare professional involvement.
• We propose a protocol to train a system that helps patients scan their joints autonomously.
• Six readers used our protocol to rate US videos, and the results show its reliability.
• This protocol represents a step toward patient-centered solutions for diagnosis of joint bleeding.

## 1 Introduction

The most frequent and severe bleeding episodes in people with hemophilia, accounting for around 80% of all bleeding episodes, occur in the joints, which can result in acute pain, reduced mobility, and long-term joint damage in the form of hemophilic arthropathy [1]. Current hemophilia management strategies emphasize the importance of early detection and treatment of joint bleeding to minimize the long-term consequences, with particular emphasis on the self-assessment of symptoms by people with hemophilia themselves [2]. In instances where a clinical evaluation is necessary, advanced imaging techniques such as ultrasound (US) or magnetic resonance imaging (MRI) may be employed to confirm a bleed [3], [4], [5], [6]. However, these are often unavailable or require specialized operators [7], [8]. The fact that bleed detection is so dependent on self-assessment by people with hemophilia introduces inaccuracies and subjectivity, since joint bleeds can be hard to distinguish from ordinary joint discomfort or arthropathy symptoms, leading to delayed or unnecessary treatment and compromising its effectiveness [9], [10], [11].

The incorporation of ultraportable US scanners designed for at-home joint assessments presents a significant opportunity to address these unmet needs in hemophilia [10], [12], as it has already been shown to be successful in other healthcare areas [13], [14]. Current telemedicine approaches require the involvement of healthcare professionals (HCPs) to guide patients through the imaging process [12], which can limit the system’s utility as a screening tool, particularly for regular assessments of all joints, as it does not allow patients the autonomy to perform routine checks independently. However, artificial intelligence (AI) and machine learning (ML) technologies have the potential to overcome this limitation [15], [16]. Several authors have shown how AI can detect joint bleeds in US images and highlight its potential use for improving US assessments, especially within applications such as automated acquisition of images, anatomical landmarking, and probe positioning guidance [17], [18], [19], [20].

We envision an innovative device system featuring an ultraportable US probe that connects wirelessly to a smartphone. Accompanied by a user-friendly mobile application, this device system would provide patients with basic visual instructions and real-time guidance for conducting blind scans of their joints [21], [22], in which the user does not have access to the live US image. The application would employ AI to assist users by providing direct feedback: specifically, a layout on the screen that visually indicates the proximity of the user to the desired scanning location while withholding direct access to US images from the patients themselves. This choice makes the envisioned system different from others in the literature, such as those described by other authors in [23], [24], and it ensures adherence to the intended use of the device; otherwise, patients might attempt to interpret the US images, leading to inaccurate conclusions regarding their own health status or mistakenly explore areas of the body unrelated to their condition, potentially complicating diagnosis and treatment.

As a first step for the development of this AI-driven system, we have created an annotation protocol to ensure accurate labeling and categorization of data with the aim of enhancing the model’s ability to guide nonprofessionals in effectively locating the correct scanning areas and acquiring a diagnostic-grade US image. While protocols such as Hemophilia Early Arthropathy Detection with Ultrasound (HEAD-US) [25] and Joint Tissue Activity and Damage Examination (JADE) [26] are validated in the medical community in hemophilia, their focus lies in clinical practice rather than AI training. Therefore, the novelty of this study lies in the proposed annotation protocol to support home implementation of US and empower people with hemophilia to monitor their joint health confidently. Given the primary purpose of training the AI algorithm, the protocol contains elements that may seem redundant in the context of regular imaging protocols.

## 2 Methods

### 2.1 Annotation protocol

#### 2.1.1 Protocol establishment

The annotation protocol was developed through an iterative process of data annotation, expert consensus, and analysis of existing clinical protocols such as HEAD-US [25] and JADE [26]. General principles for the generation of diagnostic-quality US images were applied, and the specific anatomy of the joints of interest was taken into account. This multifaceted approach ensured that the proposed protocol was grounded in established evidence while integrating practical insights from experienced practitioners.

#### 2.1.2 Projections of interest

Synovial recess distension (SRD) has been previously used as a surrogate marker for the diagnosis of joint bleeds in people with hemophilia, as it can be captured using US imaging [22], [25], [26], [27]. In contrast, specifically identifying a bleed is challenging due to the changes in echogenicity and shape over time and the lack of very specific features, in particular on still images. The detection of a distended recess alone does not establish the presence of a bleed, but it would be an important finding to initiate further evaluation. As outlined below, the change of a potentially already distended recess is also important in this context.

We identified three key projections of interest to assess SRD: the posterior recess of the elbow, the anterior subquadricipital recess of the knee, and the anterior recess of the ankle (Figure 1). These projections were selected based on their relevance to the anatomical locations where SRD is most assessed in patients with hemophilia [25], [26], [27] and provide a bidimensional view or projection of the region of interest (ROI) for each joint compared with the otherwise three-dimensional assessment that a standard US scan in a clinical setting would achieve.

**Figure 1:**
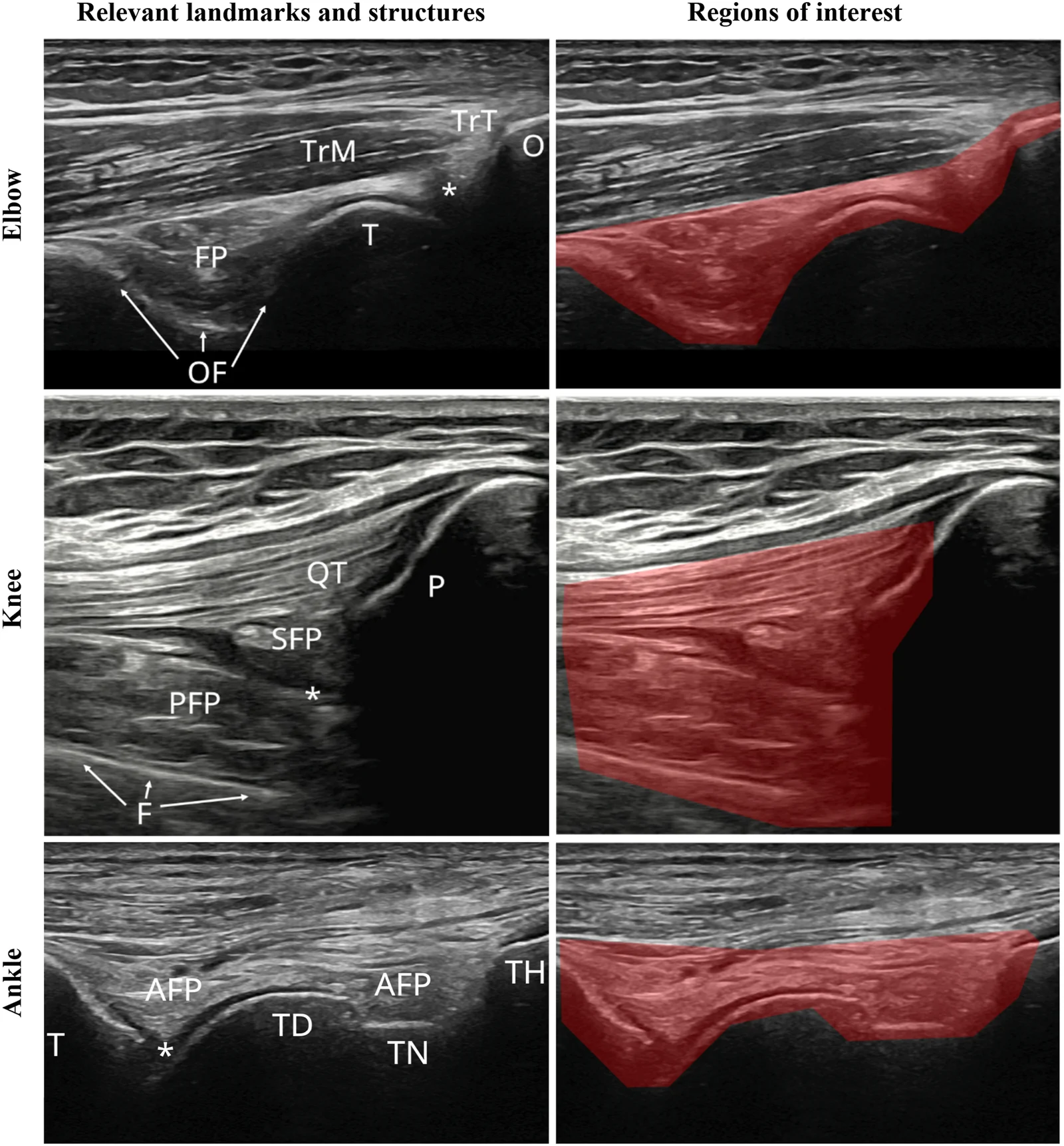
Relevant landmarks and structures in the elbow, knee, and ankle joints and regions of interest (ROI) targeted for detailed evaluation: posterior elbow projection, anterior knee projection, and anterior ankle projection. Elbow: OF, bony floor of olecranon fossa; FP, posterior fat pad; T, posterior trochlea; TrM, triceps tendon; and O, olecranon tip. The recess will distend from the right ∗ (distal) into the olecranon fossa that is proximal to ∗ and lift the fat pad. Knee: F, distal femoral shaft; PFP, prefemoral fat pad; SFP, suprapatellar fat pad; P, superior pole of the patella; and QT, quadriceps tendon. The recess will distend from the right ∗ (distal). Ankle: T, distal tibia epiphysis; AFP, anterior fat pad; TD, anterior talar dome; TN, talar neck; and TH, talar head. The recess will distend from the left ∗ (proximal) and lift the anterior fat pad.

#### 2.1.3 Image appropriateness

We define US image appropriateness as the relevance and suitability of an image for detecting SRD in the context of training AI algorithms. This distinction is important, as some criteria may not need to be assessed separately in clinical or research settings where images are evaluated by human readers.

To standardize evaluations of image appropriateness, we established eight key criteria (described in Tables 1 and 2 and Supplementary Figures 1, 2, and 3), which were derived from two dimensions: first, whether SRD can be detected in the image and, second, whether the captured projection is correct. Criteria 0, 2, 3, 4, and 5 refer to the first dimension and determine if at least part of the SRD is visible in the image and clear enough for assessment. Criteria 1, 6, and 7 refer to the second dimension and provide information on whether the joint is flexed correctly and the probe is in the right position.

**Table 1:** Image appropriateness criteria C0–C3.

Criterion	Description	Rationale
**C0—Visibility of ROI**	The ROI is fully or partially visible.	C0 ensures that the area where the recess distends is captured and at least partly visible in the image.
**C1—Visibility of bony landmarks**	The relevant parts of the bone reference points (landmarks) for each view are visible. The relevant bony structures are: bony floor of olecranon fossa (elbow), posterior trochlea (elbow), olecranon tip (elbow), distal femoral shaft (knee), superior pole of the patella (knee), distal tibia epiphysis (ankle), anterior talar dome (ankle), talar neck (ankle).	C1 ensures that relevant bony landmarks are captured in the image (as seen in Figure 1).
**C2—Clarity of bone contour**	The relevant parts of the bone contour have sufficient clarity.	C2 ensures that the bony landmarks in the image are bright and continuous to a sufficient level (Supplementary Figure 1A, Supplementary Figure 2A, Supplementary Figure 3A).
**C3—Visibility of soft tissues**	The soft tissues within the joint are visible enough throughout the ROI without significant artefacts in order to distinguish a recess distension from normal intra-articular soft tissue.	C3 ensures that the area where the recess distends is clear (Supplementary Figure 1B, Supplementary Figure 2B, Supplementary Figure 3B).

ROI, region of interest.

**Table 2:** Image appropriateness criteria C4–C7.

Criterion	Description	Rationale
**C4—Appropriateness of depth**	The image is taken with correct settings in terms of depth, ie, all relevant structures fill the image almost completely without leaving a significant black area underneath the joint (excessive depth) while at the same time not cutting off the deepest part of the joint (insufficient depth).	C4 provides a potential technical reason why criteria C1–C3 might not be fulfilled.
**C5—Appropriateness of gain**	The image is taken with the correct settings in terms of gain to depict all structures within the ROI clearly and ensure uniformity of the ROI. However, the image shall not have too much or too little gain, because it will make differentiation of soft tissues challenging.	C5 provides a potential technical reason why criteria C1–C3 might not be fulfilled.
**C6—Probe position appropriateness**	The probe is positioned to approximately acquire a midsagittal view of the joint. For the ankle, the ideal plane of acquisition is slightly more lateral than the midsagittal plane.	C6 ensures that the probe is approximately positioned along the midsagittal plane of the joint to allow more accurate longitudinal studies of the SRD progression. For the ankle, the ideal position is slightly more lateral than the midsagittal plane (Supplementary Figure 1C, Supplementary Figure 2C, Supplementary Figure 3C).
C7—Joint flexion appropriateness	The joint is at the correct degree of flexion.	C7 ensures that the joint is flexed correctly (90 degrees for the elbow, 30 to 45 degrees for the knee, and full plantar flexion for the ankle; Supplementary Figure 1D, Supplementary Figure 2D, Supplementary Figure 3D). When the flexion is not correct, the morphology of the SRD might change, and correct SRD assessment might not be performed, especially in longitudinal studies.

ROI, region of interest; SRD, synovial recess distension.

An important consideration in defining the criteria was the ability to perform longitudinal assessments of SRD, ie, to evaluate the change of the synovial recess over time. For example, if there is already a preexisting distension, an increase in this distension may indicate a new bleeding episode. To meet this requirement, criteria were needed to ensure that appropriate images are those with the probe positioned in roughly the same location, not only with regard to the mere visibility of certain bone landmarks but also with regard to being as close as possible to the midsagittal plane or slightly lateral to it for the ankle (Criterion 6). Essential for the comparison over time is also a consistent degree of flexion of the joint (Criterion 7), since it will influence the shape of the recess.

While all criteria in Tables 1 and 2 serve as critical benchmarks for image appropriateness, it is essential to acknowledge that some criteria are more objective in nature, such as C1—Visibility of bony landmarks, while others, such as C3—Visibility of soft tissues, may involve a degree of subjectivity in their assessment.

In general, when all criteria are met, SRD assessment can be performed with the highest level of confidence. However, it is important to note that SRD may still be assessed even when some criteria are not fully met. In such cases, the assessment may become more challenging, as missing information or insufficient image quality could introduce uncertainty into the evaluation process. Consequently, while failure to meet the criteria might not preclude assessment altogether, it might complicate the interpretation, necessitating a cautious approach in drawing conclusions.

#### 2.1.4 Multiclass time range selection

Assessing the appropriateness of complete US scans can be time-consuming. Our in-house pilot tests indicated that annotating a single US image according to the eight established criteria can require as much as 45 seconds. When considering a 30-second US video recorded at 27 frames per second, the total annotation time could extend to approximately 10 hours. Even annotating every fifth frame would require approximately 2 hours, which is impractical for large datasets.

For this reason, we devised a fast annotation task to obtain labeled data for training an AI model focused on providing simplified contextual guidance during blind joint scans.

To achieve this, we established a scoring scale comprising five clarity levels (L0–L4), with the intention of assigning lower scores when the ROI is not present in the US image and increasing the score as the clarity and visibility of the ROI improve. These five levels are defined based on the previously introduced appropriateness criteria (Tables 1 and 2) and are described in Table 3.

**Table 3:** ROI clarity levels.

Level	Elbow	Knee	Ankle
**L0**	No contact with skin.	No contact with skin.	No contact with skin.
**L1**	Contact with skin.	Contact with skin.	Contact with skin.
**L2**	More than half of the bony floor of the olecranon fossa is visible.	Femur is visible.	Talar dome is visible.
**L3**	All bony landmarks (bony floor of the olecranon fossa, posterior trochlea and olecranon tip) are visible.	All bony landmarks (femur and patella) are visible.	All bony landmarks (distal tip of the tibia, talar dome, talar neck) are visible.
**L4**	All bony landmarks are visible; the bone contour and soft tissues are acceptable; the olecranon tip is clearly more superficial than the posterior trochlea.	All bony landmarks are visible; the bone contour and soft tissues are acceptable; the probe is positioned in midline, and the joint is flexed correctly (30 to 45 degrees).	All bony landmarks are visible; the bone contour and soft tissues are acceptable; the probe is positioned in midline, and the joint is flexed correctly (the dome is significantly longer than the neck).

Example images for the ROI clarity levels are provided in Supplementary Figure 4.

Level L0 indicates that there is no contact between the probe and the skin, which means that the ROI is not visible at all. As the clarity of the ROI increases, the level progresses to L4, where all landmarks and anatomical features are clearly visible, suggesting optimal conditions for assessment. Examples of the ROI clarity levels are provided in Supplementary Figure 4.

This scoring scheme aids in training the AI model by providing annotated data that reflects varying degrees of ROI clarity. By employing these levels, the AI can learn to give real-time feedback during blind scanning operations, guiding laypeople toward acquiring images of sufficient quality for SRD assessment.

In general:

• In images with an L0–L1 ROI clarity level, the SRD assessment cannot be made.

• L2–L3 are transition levels, meaning that there is no guarantee that an SRD assessment can be made.

• In images with an L4 ROI clarity level, SRD assessment can be made, and both probe placement and joint flexion are optimal.

While higher scores indicate better imaging conditions, it is important to note that there may be situations where SRD can still be assessed even for images with an ROI clarity level lower than L4. However, achieving optimal ROI clarity enhances the reliability of the readings and confidence in the assessments performed.

### 2.2 POCUS2 dataset

As a sample application, the protocol was used to annotate US videos from the POCUS2 dataset. This dataset includes US videos of 100 healthy volunteers (male and female) between the ages of 19 and 70 years collected at the Novo Nordisk Clinical Innovation Centre (CLINiC) in Denmark between November and December 2024. Participant eligibility criteria are detailed in Supplementary Table 1. Further information on the study population (age, sex, height, weight, and body mass index distributions) can be found in Supplementary Figure 5. All participants provided written and verbal informed consent prior to participation. Materials used during the data collection were an iPhone 14, Clarius L7 HD3 US probe (including fan and charger), an app with predefined US probe settings for image acquisition, and US gel. The study size was determined by the available POCUS2 dataset.

During scanning, each participant positioned themselves as illustrated in Supplementary Figure 6. Afterwards, a moderator applied US gel to the area around the joint to be scanned and started the video recording. The moderator performed the scan by moving the US probe to the correct position with the help of the visual feedback provided within the app, making sure that the ROI was visible (Figure 1). Once satisfied with the image displayed on the phone screen, the moderator kept their position for 2 seconds. Finally, the moderator pressed the stop button on the app to end the recording. If necessary, due to uncertainty about having acquired the data correctly, the moderators were instructed to scan the same joint multiple times. The moderators were instructed to try to keep the whole process within 1 minute. For each participant and each joint of interest (elbow, knee, and ankle), the moderator scanned both the left and right sides of each participant.

Moderators were HCPs (eg, nurses) with a clinical background, but no previous experience in performing US scans. For this reason, prior to the study, they underwent a training session on how to perform musculoskeletal (MSK) US.

The dataset was collected and developed for internal research and development purposes. The videos are not currently intended for public release and are therefore not publicly available.

### 2.3 Data annotation

#### 2.3.1 Annotation process

A total of six readers were presented with the US video material of ankle (*n* = 201), knee (*n* = 204), and elbow (*n* = 203) joints from the POCUS2 dataset. All readers were HCPs with expertise in MSK US and related clinical and research domains. The group included five experienced sonographers and one physiotherapist with advanced research training.

Among the sonographers, several had over a decade of dedicated MSK US experience, with the majority holding formal MSK US certification. The team’s combined sonography experience spanned more than three decades, with members who were also actively involved in sonography education and research. Their backgrounds included advanced clinical scanning, instruction of sonography trainees, and prior participation in US and AI research initiatives. Additionally, the physiotherapist member held a doctoral degree and had specialized training and experience in US imaging and the management of patients with bleeding disorders.

Each reader was instructed to assign an ROI clarity level between L1 and L4 to the video time intervals with a precision of one decisecond (one-tenth of a second) based on the parameters detailed in the annotation protocol (Table 3). Requiring an explicit L0–L4 label for every decisecond would have allowed annotation omissions to be distinguished from L0 ratings but would have substantially increased the annotation burden. We therefore prioritized accurate annotation of the informative ROI clarity levels L1–L4. For a US video to be considered labeled, at least three readers had to assess and label it.

Prior to commencing the annotation task, readers participated in 1-hour online sessions every 2 weeks for a period of about 6 months, where they collaboratively discussed the annotation of individual frames from US video samples of the ankle, knee, and elbow joints sourced from entities other than POCUS2. A data arbiter supported the project to ensure data quality, protocol consistency, and adherence to study objectives. The data arbiter is a physiotherapist with a doctoral degree and over two decades of clinical and research experience in the care of individuals with bleeding disorders, with extensive clinical and academic work on the integration of point-of-care US into clinical and research settings and the development of national and international education initiatives focused on MSK US in hemophilia. The sessions had 2 main objectives: first, to identify inconsistencies in the annotation protocol through an informal review conducted by a panel independent of the protocol authors, allowing for necessary improvements; and second, to ensure that all readers were consistent in their interpretation of the protocol.

CentaurLabs (https://info.centaurlabs.com/overview) was used as the annotation platform for the task (Supplementary Figure 7).

#### 2.3.2 Annotation data preprocessing

For each reader response, time intervals in the videos that lacked any assigned ROI clarity level were operationally coded as L0.

#### 2.3.3 Annotation analysis methods

Inter-reader agreement was assessed using the intraclass correlation coefficient (ICC). ICC values range from 0 to 1 and can be classified into categories of reliability [28]: values below 0.5 indicate poor reliability, those between 0.5 and 0.75 suggest moderate reliability, values from 0.75 to 0.9 demonstrate good reliability, and values above 0.9 represent excellent reliability.

To estimate inter-reader agreement, each annotated decisecond of a video was treated as a target, or unit of analysis. Rather than treating these targets as statistically independent, linear mixed-effects models were fitted using the *lme4* package (version 2.0.6) in *R*. The models included random intercepts for participant, video, decisecond-level target, and reader. Participant and video effects accounted for cluster-level dependence among observations from the same participant and video, while the target effect represented variation shared by readers evaluating the same decisecond. The reader random effect represented systematic differences between readers and was therefore included as a source of disagreement. This provides a hierarchical, model-based analogue of the 2-way random-effects, absolute-agreement ICC [29] and permits generalization to readers similar to those included in the study.

Agreement coefficients were calculated from the estimated variance components. The variance shared by ratings of the same target was defined as the sum of the participant, video, and target variances, whereas reader and residual variances were treated as nonshared variance. ICC(2,1) quantified the expected absolute agreement of a single randomly selected reader. For the mean of a fixed number (k) of readers, the corresponding average-rating coefficient would be calculated by dividing the reader and residual variances by k, as in ICC(2, k). In our setup, however, the number of readers contributing to a target was not constant: up to six readers were available, but the observed number of ratings varied across targets. Therefore, no single integer k adequately described the reliability of the observed target averages. For descriptive purposes, an effective number of readers ($k_{\text{eff}}$) was calculated as the harmonic mean of the target-specific rating counts. Consequently, $k_{\text{eff}}$ does not need to be an integer and should not be interpreted as the number of readers who evaluated every target. The average-rating coefficient, which we refer to as ICC(2, $k_{\text{eff}}$), is expected to exceed ICC(2,1) whenever more than one rating contributes to an average because averaging reduces the contribution of reader and residual disagreement.

Separate models were fitted for the ankle, elbow, and knee, and an additional pooled model included joint as a fixed effect to adjust for systematic differences in mean ratings among joints while assuming common variance components across joints. As a sensitivity analysis, the models were repeated after excluding each reader individually, with $k_{\text{eff}}$ recalculated after every exclusion. These analyses were interpreted as reader-influence diagnostics. Ninety-five percent confidence intervals were obtained using a parametric bootstrap with 1000 replicates. The interval limits were defined by the 2.5th and 97.5th percentiles of the bootstrap estimates.

## 3 Results

In Figure 2, we present the distribution of annotations categorized by joint. Based on the data from the POCUS2 study, the most represented ROI clarity levels (determined by floor-rounded median-derived aggregate label) are L3 and L4, accounting altogether for 79%, 85%, and 67% of the elbow, knee, and ankle data, respectively. Notably, very few deciseconds of knee videos were labeled as L1. This scarcity is attributed to the fact that when there is skin contact between the knee and the US probe, the femur almost certainly appears in the image, resulting in at least an L2 ROI clarity level. Additionally, an overall agreement among readers can be observed on how to annotate the data according to our protocol. Specifically, for the majority of time units, at least two readers agreed on the annotation, and for the knee joint, 62% of the cases showed unanimous agreement among all readers. We also compared the annotations of each reader with the floor-rounded median-derived aggregate label, represented in confusion matrices in Supplementary Figures 8–10 for elbow, knee, and ankle, respectively. As expected, the analysis reveals that readers generally align with the floor-rounded median-derived aggregate label, and, in case of disagreement, they typically annotate a time unit with a neighboring ROI clarity level.

**Figure 2:**
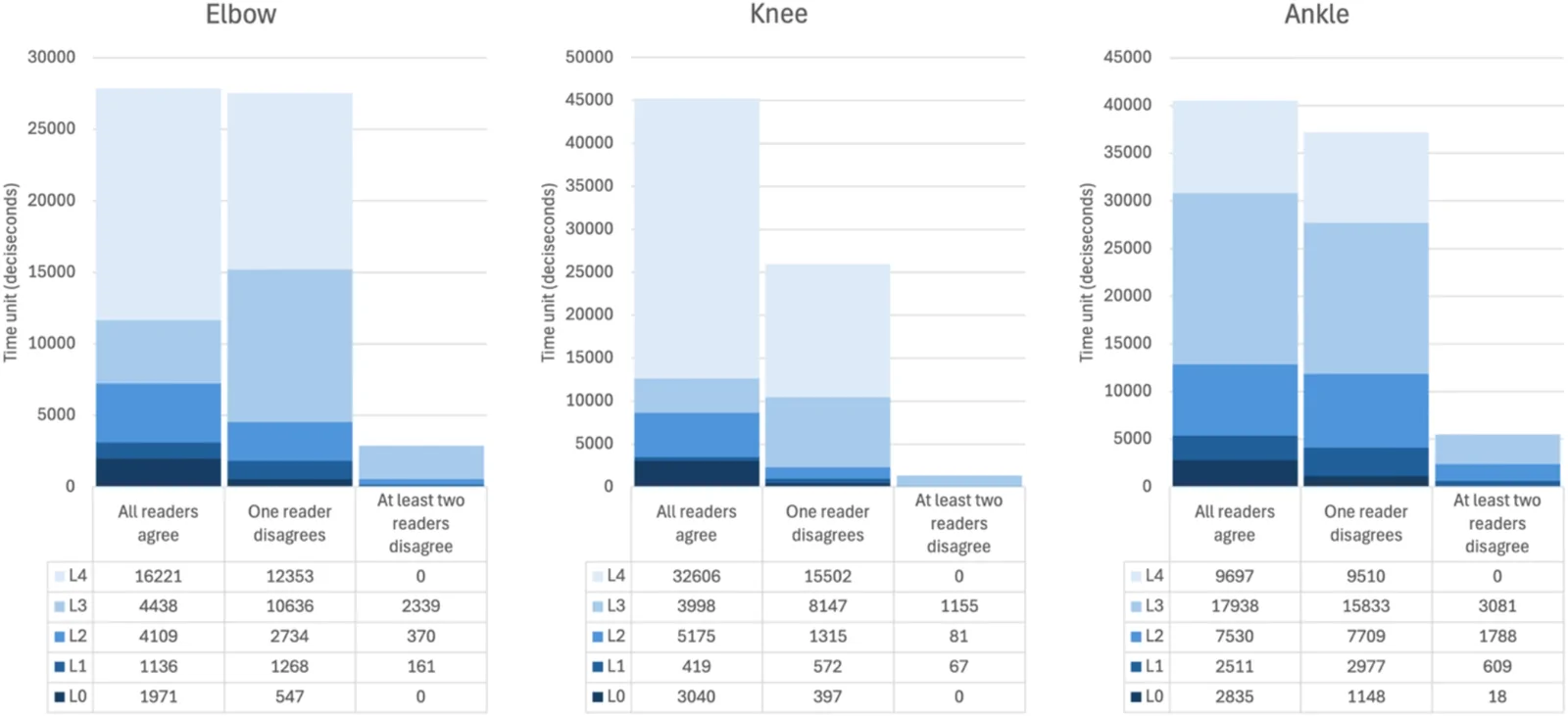
Distribution of the units of analysis (decisecond units) based on whether all readers agree, one of them disagrees, or all of them disagree. The clarity levels (L0–L4) indicate the floor-rounded, median-derived aggregate label.

A more accurate analysis of the level of agreement between the annotators is also presented in terms of ICC in Table 4. The ICC values for the full dataset across all joints yielded an ICC(2,1) of 0.7643 (95% confidence interval [CI]: 0.7511–0.7732), indicating a good level of agreement among the six readers. When examining the specific joints, the knee demonstrated the highest reliability with an ICC(2,1) of 0.8437 (95% CI: 0.8323–0.8507), followed by the ankle at 0.7364 (95% CI: 0.7175–0.7513) and the elbow at 0.7109 (95% CI: 0.6662–0.7389). The ICC values also reveal stronger agreement when considering average measures (ICC(2, $k_{\text{eff}}$)), with the full dataset recording an ICC of 0.9075 (95% CI: 0.9013–0.9116). This trend continues with the knee joint, presenting the highest ICC(2, $k_{\text{eff}}$) at 0.9420 (95% CI: 0.9373–0.9449). Excluding individual readers from the pool reveals a slight decrease in the ICC for readers 377101, 377693, and 378277, suggesting that these readers are the most aligned with the majority in their annotations.

**Table 4:** Intraclass correlation coefficient (ICC) for agreement on the full dataset (all joints) and different subsets (only ankle, only elbow, only knee, or all joints excluding individual readers).

Dataset	Number of time units	$k_{\text{eff}}$	ICC(2,1) (95% CI)	ICC(2, $k_{\text{eff}}$) (95% CI)
All joints	213,941	3.026	0.7643 (0.7511–0.7732)	0.9075 (0.9013–0.9116)
Ankle	83,184	3.056	0.7364 (0.7175–0.7513)	0.8952 (0.8859–0.9023)
Elbow	58,283	3.002	0.7109 (0.6662–0.7389)	0.8807 (0.8570–0.8947)
Knee	72,474	3.010	0.8437 (0.8323–0.8507)	0.9420 (0.9373–0.9449)
Excl. reader 377090	213,941	2.538	0.7804 (0.7662–0.7891)	0.9002 (0.8926–0.9047)
Excl. reader 377101	213,941	2.098	0.7407 (0.7269–0.7502)	0.8570 (0.8481–0.8630)
Excl. reader 377587	213,941	2.706	0.7717 (0.7534–0.7826)	0.9014 (0.8921–0.9069)
Excl. reader 377693	213,941	2.482	0.7618 (0.7435–0.7721)	0.8881 (0.8780–0.8937)
Excl. reader 378277	213,941	2.319	0.7413 (0.7265–0.7504)	0.8692 (0.8603–0.8747)
Excl. reader 379997	213,941	2.482	0.7798 (0.7664–0.7879)	0.8979 (0.8906–0.9021)

## 4 Discussion

The proposed annotation protocol specifies eight appropriateness criteria used to derive a five-level scoring system applied by six readers to rate MSK US videos of healthy subjects. Several factors may reduce inter-reader agreement. First, readers assessed full decisecond-resolution videos rather than still frames, exposing evaluations to motion blur and other artifacts that complicate frame interpretation and are only partially controllable by protocol. Second, there is a trade-off between accuracy and speed: the five-level ROI clarity score streamlines assessment relative to an exhaustive eight-criterion frame-by-frame evaluation, but annotating every decisecond remains time-consuming. Readers were therefore asked to balance precision with efficiency, which may have produced variability in strictness across individuals. Finally, some criteria are inherently subjective (eg, soft-tissue appropriateness in L4 versus clear bony landmarks in L2), fostering divergent interpretations and reducing annotation consistency.

Despite these potential sources of error, the overall distribution of annotations shows that the majority of annotators tend to agree on how to annotate the data, with knee videos exhibiting the highest level of agreement. These findings are further confirmed by the ICC analysis, where the good ICC(2,1) value of 0.7643 for the overall dataset indicates a reliable level of inter-reader agreement. The high ICC(2,1) value of 0.8437 for the knee suggests that the images from this joint are the easiest to interpret, likely due to the low variability in anatomical positioning and probe placement during acquisition. The substantial increase in reliability when transitioning from individual to average ratings among readers (with an ICC(2, $k_{\text{eff}}$ of 0.9075 for the full dataset) emphasizes the advantage of aggregating annotations. Given this finding, we recommend employing an annotation process that promotes the use of multiple readers rather than relying solely on single-reader annotations. The slight decreases in ICC observed when excluding specific readers—namely, those numbered 377101, 377693, and 378277—suggest that these individuals’ annotations are most closely aligned with the majority opinion. However, the small magnitude of the decrease indicates that there were no clear outliers among the readers. If the decrease had been larger, it could have highlighted the need for further training or calibration efforts.

## 5 Conclusion

The proposed annotation protocol developed in the context of an AI-driven guidance system for blind joint US scans, where blind refers to the fact that the user will not have live access to the US images during the scan, represents a potentially significant advancement in facilitating assessments in a home environment for people with hemophilia. However, there are some notable limitations that must be addressed in future research.

Since our annotation workflow did not distinguish an intentionally unlabeled interval representing L0 from a true annotation omission, intervals operationally coded as L0 may correspond either to genuine L0 observations or to omitted annotations. Aggregating annotations from multiple readers may mitigate the effect of occasional reader-specific omissions, although it cannot eliminate the possibility that all readers omitted the same interval. This multi-reader aggregation is an intended part of the annotation workflow because the resulting labels are expected to be used as reliable training labels for machine-learning models, rather than relying on labels from a single reader.

We recognize that the current guidance of the AI-driven device system we envision is a simplified overview of how close users are to the anatomical landmarks of interest, yet the actual process of performing a scan is complex and multifaceted. Factors such as joint positioning and flexion, probe placement (medial, midline, lateral, proximal, and distal), probe tilting, and the variation in probe pressure (evenly applied across the entire probe head versus uneven tilting techniques) all play critical roles in the quality of the US image obtained. To better understand how users interact with the simplified guidance, conducting usability studies will be essential. These studies should investigate how effectively patients can navigate the various dimensions of probe manipulation while relying on the simple direct feedback provided by the guidance system.

We also acknowledge that certain situations may challenge the AI-driven guidance system. These could include situations in which the user has limitations in their ability to flex their joints, a relatively common issue in people with advanced hemophilia, or extreme osseous changes in the joint (eg, giant osteophytes in the ankle).

It is important to note that this study did not evaluate the diagnostic accuracy of US or the ability of the annotation framework to diagnose hemarthrosis. The dataset consisted of US videos acquired from healthy volunteers by healthcare professionals and did not include self-acquired videos of patients with clinically confirmed joint bleeding or an independent diagnostic reference standard. Future studies should evaluate the framework using data from people with hemophilia and should assess whether AI models trained with these annotations can effectively guide patients during self-directed joint US scans. The diagnostic accuracy of such systems for hemarthrosis also requires separate clinical validation against an appropriate reference standard.

In summary, while our findings represent a promising step forward in empowering patients to monitor their joint health independently, recognizing these limitations is critical for refining the technology and ensuring its effectiveness in real-world applications for people with hemophilia. Future research should focus on addressing these limitations to enhance the robustness, usability, and clinical impact of our proposed solution.
