# Supplementary material for: Enabling artificial intelligence-driven blind joint ultrasound self-scans in hemophilia: an annotation protocol
Source: Res Pract Thromb Haemost. 2026 Oct 13;10(7):107019. doi: 10.1016/j.rpth.2026.107019 (PMC13760104; doi:10.1016/j.rpth.2026.107019)
Supplement: Supplementary file 1 [file mmc1.pdf]

Supplementary Table 1. POCUS2 participant eligibility criteria

Inclusion criteria	Exclusion criteria
• Male and female adults (≥ 18 years old at the time of inclusion)<br>• BMI ≥ 18.5 kg/m^2<br>• Able to read and understand written English	• Participants with prosthetic joints.<br>• Participants with joints that have undergone major orthopaedic surgery (participants that have undergone minor surgeries such as arthroscopy of the knee can be included).<br>• Participants with clinically significant disease of the joints.

BMI: Body mass index
