# Supplementary material for: Enabling artificial intelligence-driven blind joint ultrasound self-scans in hemophilia: an annotation protocol
Source: Res Pract Thromb Haemost. 2026 Oct 13;10(7):107019. doi: 10.1016/j.rpth.2026.107019 (PMC13760104; doi:10.1016/j.rpth.2026.107019)
Supplement: Supplementary file 2 [file mmc2.pdf]

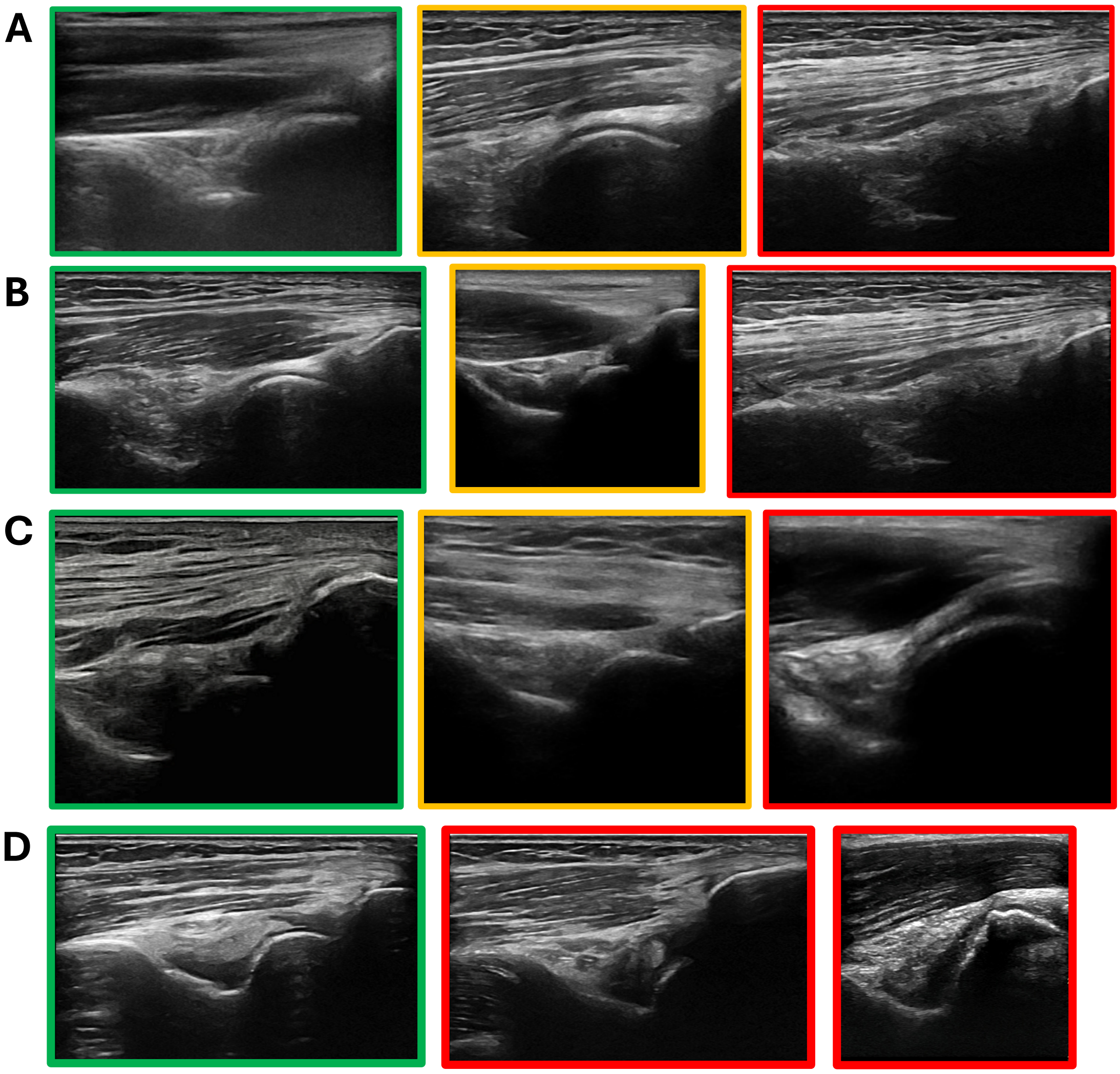

Supplementary Figure 1. Examples of acceptable (green border), borderline acceptable (yellow border) and not acceptable (red border) images of the elbow joint based on image appropriateness key criteria: (A) C2 – clarity of bone contour, (B) C3 – visibility of soft tissues, (C) C6 – Midline probe position, and (D) C7 – joint flexion.
