# Supplementary material for: Enabling artificial intelligence-driven blind joint ultrasound self-scans in hemophilia: an annotation protocol
Source: Res Pract Thromb Haemost. 2026 Oct 13;10(7):107019. doi: 10.1016/j.rpth.2026.107019 (PMC13760104; doi:10.1016/j.rpth.2026.107019)
Supplement: Supplementary file 7 [file mmc7.pdf]

	Top View	Side View
**Elbow**	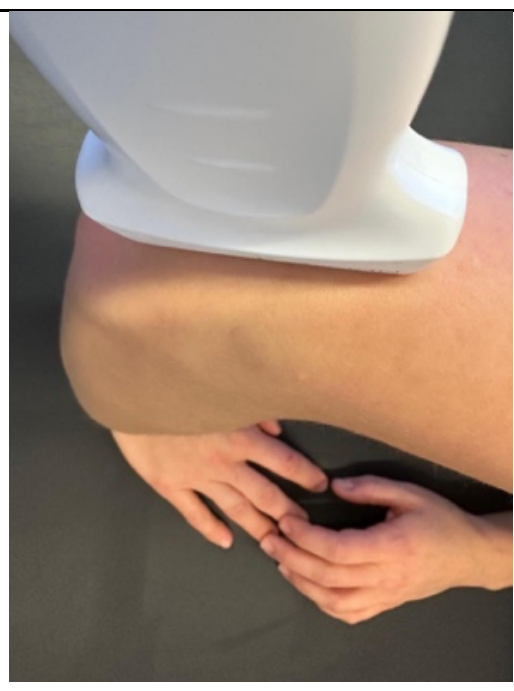	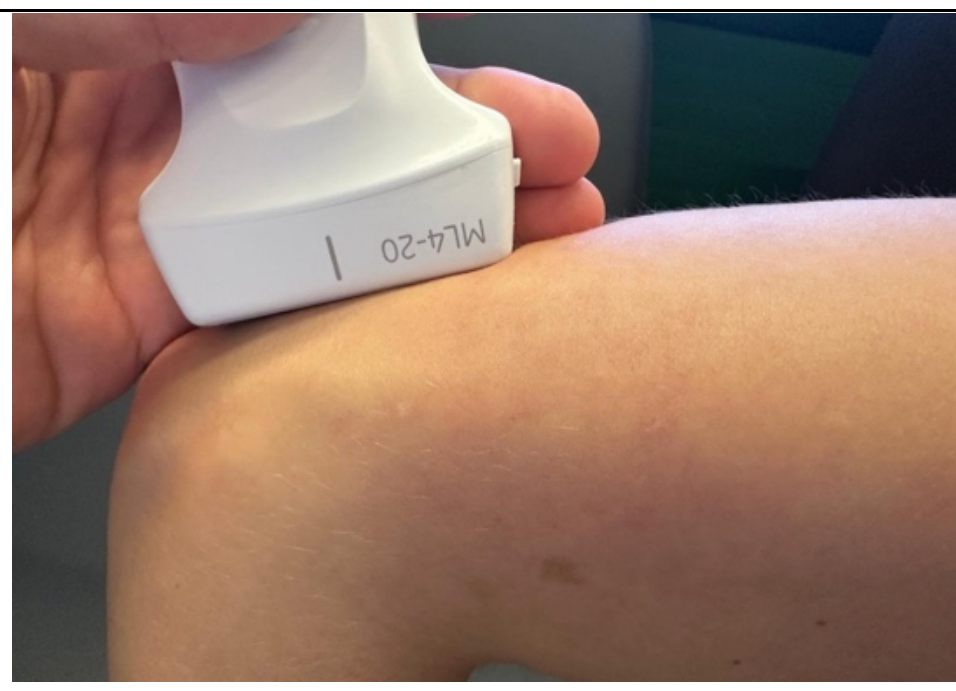
**Knee**	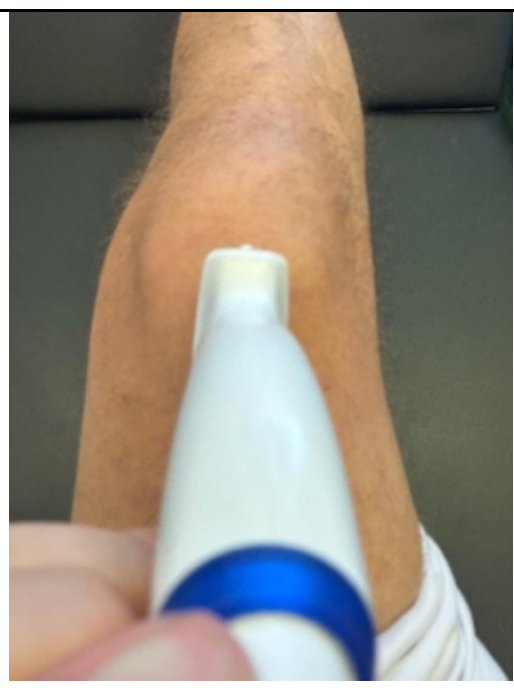	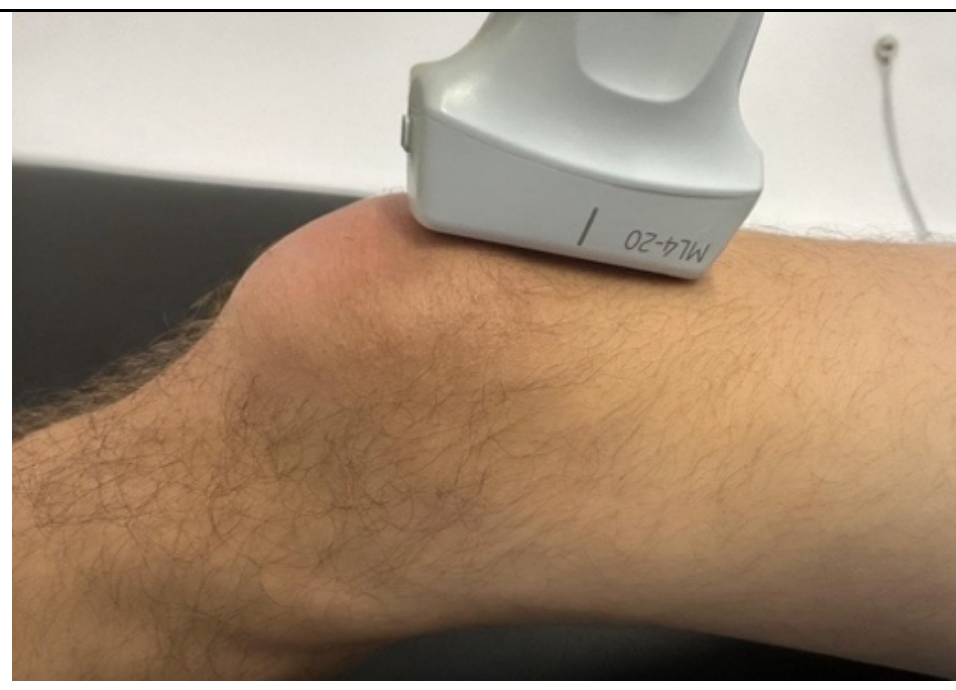
**Ankle**	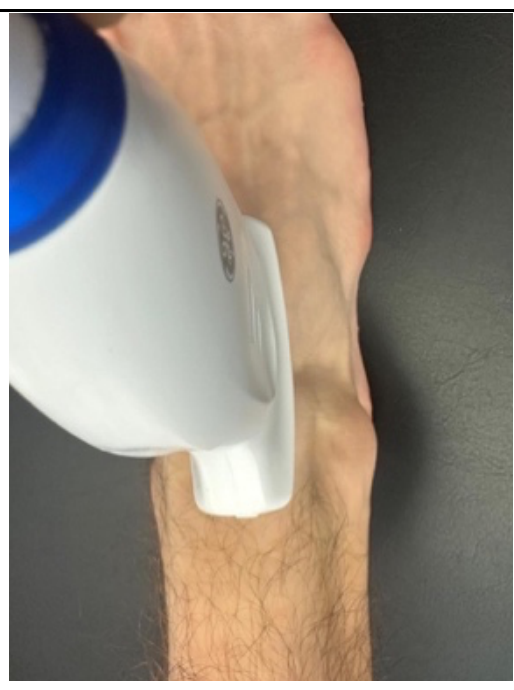	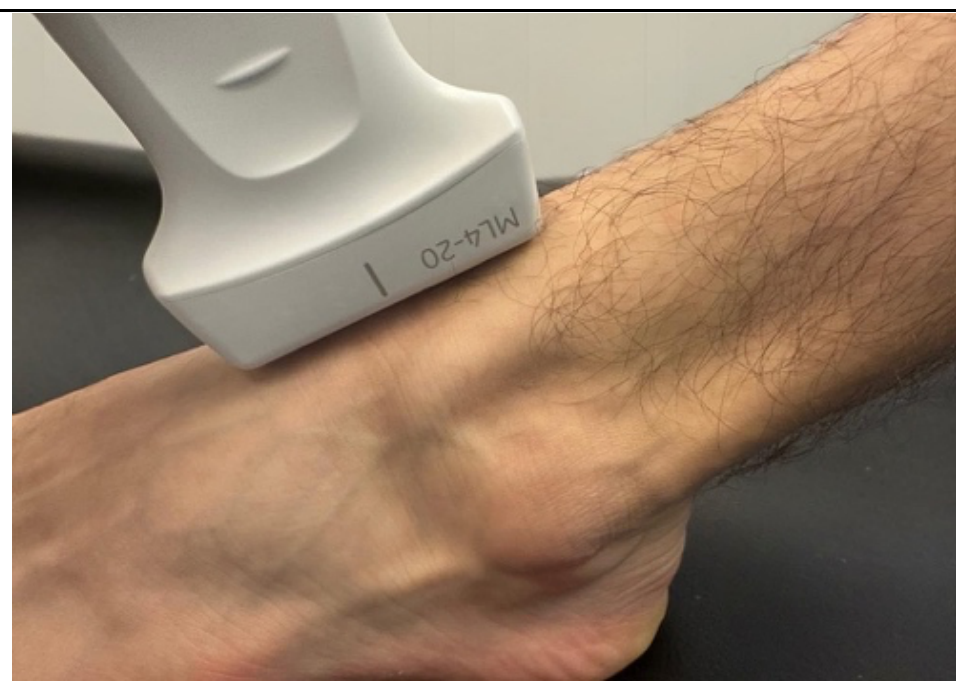

Supplementary Figure 6. Top and side view scanning positions for elbow, knee and ankle.
