# Supplementary material for: Enabling artificial intelligence-driven blind joint ultrasound self-scans in hemophilia: an annotation protocol
Source: Res Pract Thromb Haemost. 2026 Oct 13;10(7):107019. doi: 10.1016/j.rpth.2026.107019 (PMC13760104; doi:10.1016/j.rpth.2026.107019)
Supplement: Supplementary file 8 [file mmc8.pdf]

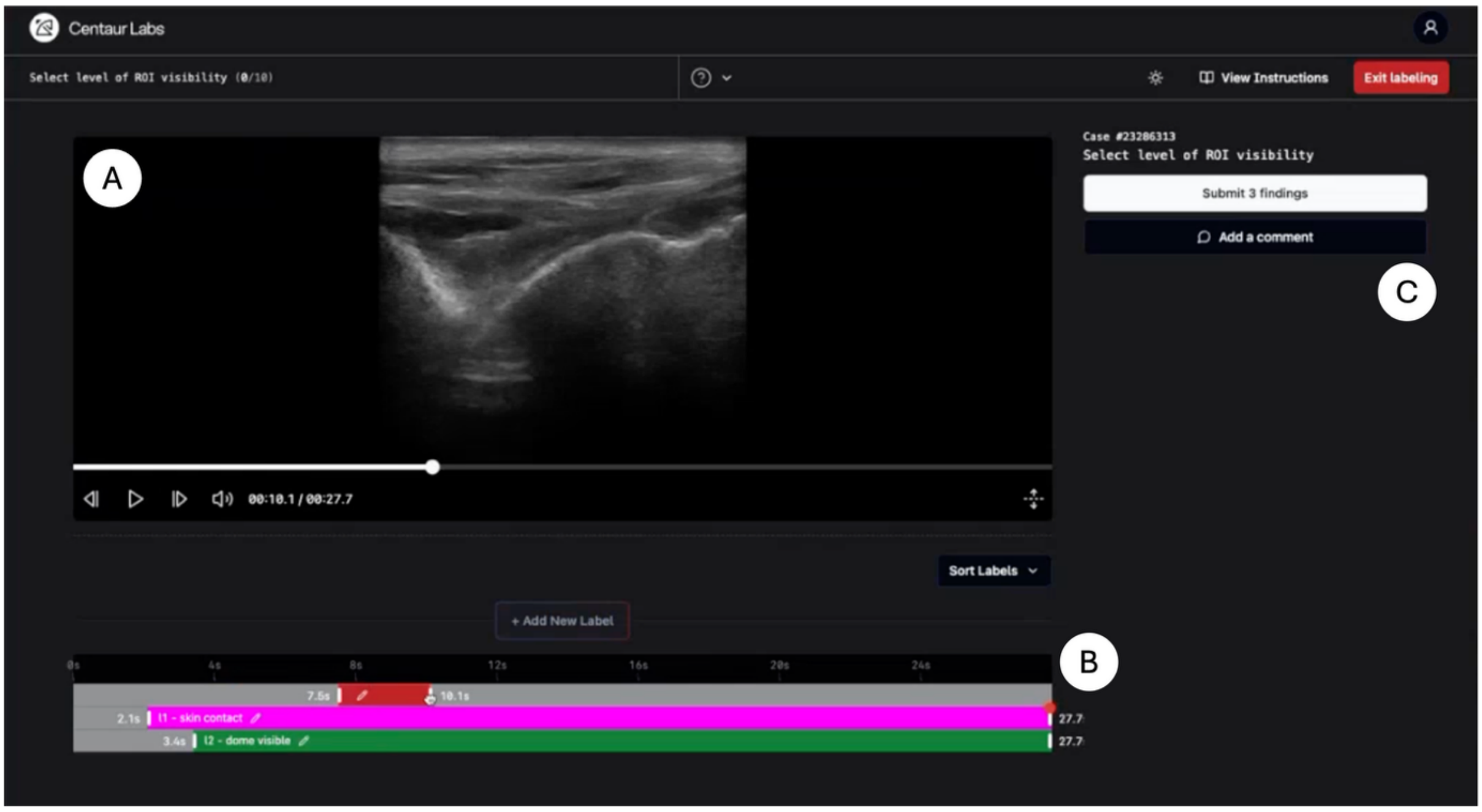

Supplementary Figure 7. CentaurLabs platform UI. A) Video player, where the ultrasound video appears. B) Time range selection, where the readers can select the ROI clarity level for a specific time range. C) Submission and comment buttons, used by readers to submit their annotations and/or leave comments about the video.
