# Supplementary material for: Enabling artificial intelligence-driven blind joint ultrasound self-scans in hemophilia: an annotation protocol
Source: Res Pract Thromb Haemost. 2026 Oct 13;10(7):107019. doi: 10.1016/j.rpth.2026.107019 (PMC13760104; doi:10.1016/j.rpth.2026.107019)
Supplement: Supplementary file 9 [file mmc10.docx]

Supplementary Figure 9. Confusion matrices for the knee.
